# Iontophoresis-Infused Deep Topical Anesthesia and Injectable Local Anesthesia for Dental Procedures Among Pediatric Patients: Performances and Consequences

**DOI:** 10.7759/cureus.43748

**Published:** 2023-08-19

**Authors:** Harsh Mistry, Shobha Fernandes, Md. Ahsanul Haq, Yash Bafna, Rohan Bhatt, Susmita Sinha, Shreya Gajjar, Santosh Kumar, Mainul Haque

**Affiliations:** 1 Pediatric and Preventive Dentistry, Karnavati School of Dentistry, Karnavati University, Gandhinagar, IND; 2 Pediatric and Preventive Dentistry, Narsinhbhai Patel Dental College and Hospital, Sankalchand Patel University, Visnagar, IND; 3 Bio-Statistics, Infectious Diseases Division, International Centre for Diarrhoeal Disease Research, Bangladesh (icddr,b), Dhaka, BGD; 4 Pediatric Dentistry, Karnavati School of Dentistry, Karnavati University, Gandhinagar, IND; 5 Physiology, Khulna City Medical College and Hospital, Khulna, BGD; 6 Periodontology, Karnavati School of Dentistry, Karnavati University, Gandhinagar, IND; 7 Periodontology and Implantology, Karnavati School of Dentistry, Karnavati University, Gandhinagar, IND; 8 Karnavati Scientific Research Center (KSRC), Karnavati School of Dentistry, Karnavati University, Gandhinagar, IND; 9 Pharmacology and Therapeutics, National Defence University of Malaysia, Kuala Lumpur, MYS

**Keywords:** consequences, performances, children, dental procedures, injectable local anesthesia, deep topical anesthesia, needleless anesthesia, topical anesthesia, iontophoresis

## Abstract

Introduction: Exploring routes of needle-free anesthesia has drawn particular attention to the iontophoretic technique. Iontophoresis has a wide range of applications in dentistry, treating hypersensitivity, oral ulcers, non-invasive procedures of deep topical anesthesia, etc. Hence, this research was performed for a comparative assessment of topical anesthesia spray infused via iontophoresis and local anesthesia (LA) infiltration for dental procedures among 5-12-year-old patients.

Materials and methods: A split-mouth, randomized clinical trial was undertaken over two years among study subjects aged 5 to 12 years. They were randomly assigned to one of two groups: the first (Group A - iontophoresis group) received topical anesthesia spray (Lidayn®; Pyrax Polymers, Roorkee, India) applied by iontophoresis, and the second (Group B - LA infiltration group) received local infiltration of 2% lignocaine solution (LignoTer®; Lusture Pharma, Ahmedabad, India), where primary teeth extraction or pulpectomy was performed. The Wong-Baker Facial Pain Rating Scale (WBFPRS) was used for a subjective assessment immediately following anesthesia.

Results: The mean value of current intensity for the extraction procedure was 9.43±0.95 mA, and the duration of application was 1.85±0.80 minutes. The mean value of current intensity for pulpectomy was 9.07±1.34 mA, and the time was 2.40±0.74 minutes. In inter-group comparison, WBFPRS scores were lower in Group A (1.96±1.64) compared to Group B (3.62±1.11), which was statistically significant with p=0.001.

Conclusion: Compared to local infiltration, iontophoresis as a non-invasive approach for topical anesthesia was more well-received by pediatric patients.

## Introduction

Children are frequently reluctant to receive dental treatment due to pain-related fear and anxiety. Local anesthesia (LA) is one of the most effective pain management techniques. However, the LA administration method, namely injection, can generate anxiety and panic. Various strategies can reduce the pain and discomfort caused by injections. These include suitable behavior management approaches, adjusting the pH and temperature of the anesthetic solution, and administering the solution at a slower rate [1]. Other alternative methods are electronic dental anesthesia, intra-oral lignocaine patch: Dentipatch, jet injection, iontophoresis, eutectic mixture of local anesthetics, computer-controlled local anesthetic delivery devices, and intra-osseous systems. All the advances mentioned above deposit LA solution with the help of a needle except iontophoresis. Verratti is credited with being the first person to describe the iontophoresis principle in 1747; nevertheless, it was not until 1900 that LeDuc was the first to popularize its use in the medical field [2]. Since its invention in 1900, iontophoresis has found widespread application in the medical community for transdermal drug administration [3].

Iontophoresis has a wide range of applications in dentistry such as tooth decalcification and hypersensitivity. After topical administration, it can transfer LA to deeper tissues. It facilitates the penetration of positively charged substances into tissues affected by electrical charges, such as lignocaine and adrenaline. This method might provide better patient care and enhance patient-dentist interaction by avoiding needle use [4]. The total dose delivered (in milliamperes-minute) is proportional to the A comparative assessment of deep topical anesthesia (with 15% lidocaine topical spray (Lidayn®)) infused via iontophoresis and local anesthesia (infiltration of 2% lignocaine solution (LignoTer®; Lusture Pharma, Ahmedabad, India)) for dental procedures among 5-12-year-old patients, product of current (in milliamperes), and application time (in minutes) [3].

Lidocaine iontophoresis, extensively researched for transdermal usage in surgical procedures, has primarily been examined for trans-buccal delivery in combination with penetration enhancers. This is because transdermal administration of lidocaine was found to be ineffective. When it comes to developing an effective drug delivery system for topical deep anesthesia, however, a few challenges need to be surmounted first. The system needs to be able to deliver the medication promptly, keep the drug in close contact with a predetermined location, target the release and permeation of the medication via the mucosa, induce anesthesia, maintain it for the proper amount of time, and make it easy to remove after the surgery [5].

Recent clinical research on iontophoresis's potential dental applications is scarce. Needle-free drug delivery systems, which could be made possible using this method, are mostly ignored in dental clinical practice [4]. Therefore, researchers looked at how well 2% lignocaine solution infiltration and 15% lidocaine topical spray infused by iontophoresis performed during dental treatments on children aged 5 to 12 years.

Aim and objectives

A comparative assessment of deep topical anesthesia (with 15% lidocaine topical spray (Lidayn®)) infused via iontophoresis and LA (infiltration of 2% lignocaine solution (LignoTer®)) for dental procedures among 5-12-year-old patients.

The primary objective of this study was to determine the current intensity of iontophoresis application with length of time, and whether or not deep topical anesthesia was adequate for routine dental procedures. The secondary objective was to determine the patient's comfort and acceptance level.

## Materials and methods

This was a split-mouth, crossover comparative study.

Source of data

Children aged 5 to 12 years, irrespective of race, sex, religion, and socio-economic status, who are indicated for bilateral extraction or pulpectomy of primary teeth were selected from the outpatient department of Paediatric and Preventive Dentistry, Narsinhbhai Patel Dental College & Hospital, Visnagar, Gujarat, India. Additionally, the age and gender of the participating patients in both groups were the same because the same patient had undergone two different modalities. The data were also the same for both groups, which provides us with the actual comparison between two groups/two other modalities.

Sample size estimation

The sample size was calculated using the formula: Sample size= 2*(Z-alfa/2 +Z1-beta) ^2/ (m1-m2/sigma) ^2 where sigma=1.1 Standard deviation. m1= 1.96 (one group mean). m2= 2.62 (second group mean) where m denotes the mean of each group, and sigma represents the standard deviation. The sample size obtained was 78. We kept it at 80, which was calculated with an accepted minimum possible alpha error of 0.05, the power of the study was 95%, and the effect size was 0.36.

**Figure 1:**
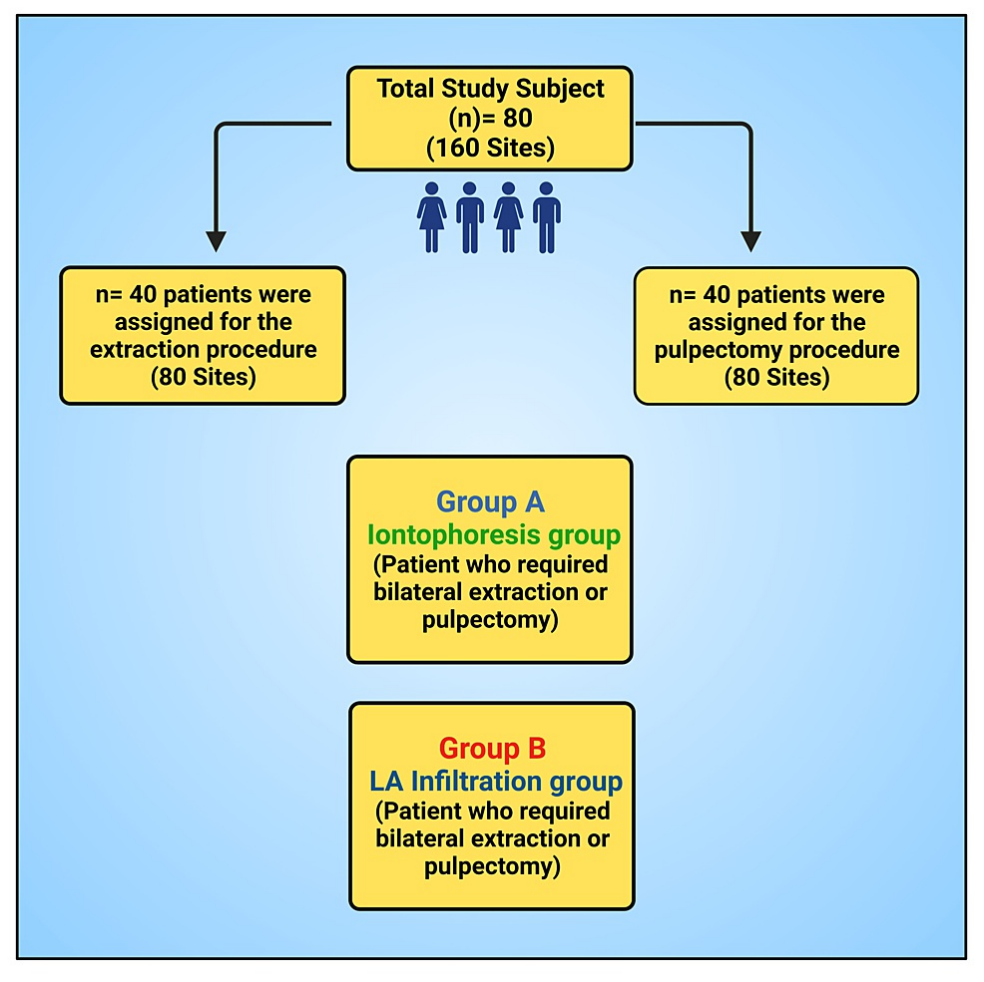
A flow chart illustrating the study group This figure has been drawn utilizing the premium version of BioRender with license number SZ25MUVO0V. Image Credit: Susmita Sinha LA: Local anesthesia

A total of 80 study subjects in the age group of 5-12 years who required bilateral extraction or pulpectomy of primary teeth for the first time were included in the study. Forty patients (80 sites) were assigned to the extraction procedure, and 40 patients (80 sites) to the pulpectomy procedure. A patient who required bilateral extraction or pulpectomy was randomly assigned to the iontophoresis group (Group A) and LA infiltration group (Group B) by the lottery method (Figure 1).

Inclusion criteria of this study were patients undergoing treatment for the first time, those additionally requiring bilateral extraction or pulpectomy of primary teeth, and those who exhibit cooperative behavior. Furthermore, those patients demonstrated competency in using the Wong-Baker Facial Pain Rating Scale (WBFPRS) and were aged between 5 and 14 years. Exclusion criteria were those patients with known drug allergies, pre-existing debilitating systemic or metabolic diseases, dental abscess or local infection, cardiac pacemaker, and metal prosthesis. The study was conducted from November 2017 to November 2019 (two years).

The populations under study were split into two distinct groups. In group A, 15% lidocaine topical anesthetic spray (Lidayn® - Pyrax Polymers, Roorkee, India) was applied on the previously dried buccal and lingual mucosal surface, followed by the application of the anode tip of Iontophoresis equipment/unit. At the same time, the patient held the cathode to complete the circuit for 1 min application each on both buccal and lingual surfaces comprising one cycle. Researchers strictly maintain iontophoresis guidelines. Therefore, they can conduct the procedure correctly. The maximum tolerated intensity of current was measured in mAmp, and the duration of application was measured in minutes. After the application of three cycles, if anesthesia was not achieved, injectable LA was administered.

In Group B, topical anesthesia spray was followed by the standard technique for infiltration anesthesia. Subjective assessment for dental anxiety was performed by WBFPRS, followed by clinical procedures of extraction/pulpectomy.

Ethical approval

This study obtained ethical approval from the Institutional Review Board of the Faculty of Dental Science, Sankalchand Patel University Visnagar 384315, Gujarat, India. Dr. J R Patel, Member Secretory, Ethical Committee Review Board Visnagar, signed the Approval. The ethical approval certificate is uploaded as Appendix-1 as it does not contain any reference number. Additionally, all patients and their guardians were clearly briefed about the study plan and future scientific publication. The written informed consent was obtained before any intervention was conducted before ethical approval certificate was issued. Nevertheless, we started paperwork and planned the research before certification was obtained with due permission of the committee. 

Statistical analysis plan

To examine the demographic characteristics (age and sex) of the local anesthetic and iontophoresis groups, chi-square tests were performed for categorical variables. At the same time, independent sample t-tests were used for continuous variables. The association between the local anesthetic and iontophoresis groups and the WBFPRS score was assessed using independent sample t-tests and multivariate regression analysis. In the multivariate regression analysis, age and sex were included as covariates.

A multiple linear regression model was employed to explore the relationship between the procedure types (extraction and pulpectomy) within the Iontophoresis group and the current intensity and duration, incorporating all relevant covariates in the same model. Statistical comparisons were conducted using STATA-15 software, while graphical presentations were created using GraphPad Prism 8.3.2. A significance level of p<0.05 was used to determine statistical significance. 

## Results

This study was conducted with 80 participants, and the average age of both groups was 9.41±2.35 years (Table 1). Subsequently, the iontophoresis and LA infiltration groups were stratified by the extraction and pulpectomy group that consisted of 40 patients in each group. The average age of the patients in the extraction group was 10.13 ± 2.29 years, while the pulpectomy group had an average age of 8.93±2.46 years (Table 2). It is worth noting that the pulpectomy group's mean age was significantly higher than that of the extraction group.

**Table 1:** Age and gender distribution for both the groups Notes: Data were presented as mean±SD or number with percent in the parenthesis—chi-square for categorical values, and an independent sample t-test was used to estimate the p-value for continuous data.

	Local anesthetic (n=80)	Iontophoresis (n=80)	p-value
Sex			
Male	41(51.2%)	41(51.2%)	0.999
Female	39(48.8%)	39(48.8%)	
Age, years	9.41±2.35	9.41±2.34	0.999

**Table 2:** Distribution of age and sex of subgroup extraction and pulpectomy group Notes: Data were presented as mean±SD or number with percent in the parenthesis. The Chi-square test was used for categorical values and a continuous data independent sample t-test was used to estimate the p-value.

	Local anesthetic (n=80)			Iontophoresis (n=80)		
	Pulpectomy (n=40)	Extraction (n=40)	p-value	Pulpectomy (n=40)	Extraction (n=40)	p-value
Sex						
Male	22(55.0%)	19(47.5%)	0.655	22(55.0%)	19(47.5%)	0.655
Female	18(45.0%)	21(52.5%)		18(45.0%)	21(52.5%)	
Age, years	10.1±2.28	8.70±2.22	0.006	10.1±2.28	8.70±2.22	0.006

**Figure 2:**
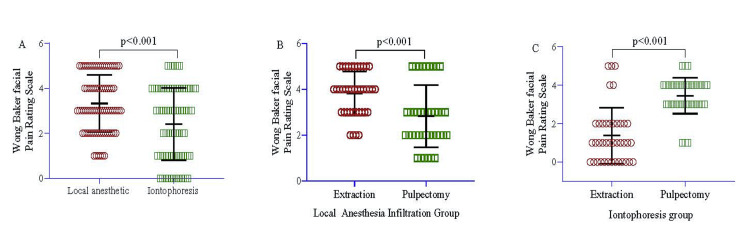
Comparison between local anesthetic and iontophoresis (A) and intra-group comparison of extraction (B) and pulpectomy group (C). An independent sample t-test was used to estimate the p-value.

In the local anesthetic and iontophoresis group, the WBFPRS was measured to be 3.32±1.27 and 2.41±1.60, respectively. This indicates that the local anesthetic group had a score that was 0.91 units higher than that of the iontophoresis group, and this difference was statistically significant (95% CI=0.46, 1.36, p<0.001) (Figure 2A). When examining the local anesthetic and iontophoresis group based on the type of procedure, the extraction procedure in the local anesthetic group had a WBFPRS mean score of 3.83±0.96. In contrast, the pulpectomy procedure had a score of 3.07±1.34. After accounting for age and sex in the regression model, it was found that the extraction procedure had a score that was 2.12 units higher compared to the pulpectomy procedure, and this difference was statistically significant (95% CI=1.58, 2.65, p<0.001) (Figure 2B). Interestingly, the WBFPRS score in the iontophoresis group showed a different pattern. In this group, the extraction procedure had a significantly lower score of 0.98 units compared to the pulpectomy procedure (95% CI=-1.51, -0.46, p<0.001) (Figure 2C).

In the multiple regression model, a higher duration was noted in the pulpectomy group by 0.35 min (95% CI=0.03, 0.67, p=0.035) compared to the extraction procedure. The duration showed a significant decrease of 8% when age was increased by one year (95% CI=-0.15, -0.01, p=0.025) (Table 3). An independent t-test was applied for the mean current intensity value, which was 9.43 ± 0.95 mA for the extraction procedure; for the pulpectomy procedure, the mean value was 9.07 ± 1.34 mA. The majority, 81.8%, of individuals received the current intensity between 9 mA and 10 mA (Table 4). The mean value for the duration of the application of cycles was 1.85 + 0.80 min (1.85 + 0.80 x 2 = 4 min) for the extraction procedure. For the pulpectomy procedure, the mean value of cycles was 2.40 + 0.74 min (2.40 + 0.74 x 2 = 6 min). Twenty six out of 55 patients (45%) required two cycles (4 minutes) for the extraction procedure and three cycles (6 minutes) for the pulpectomy procedure (Table 4). Age has been incorporated into the regression model, revealing that only age exhibits a notable impact on the application duration. Specifically, when age increased by one year, there was a substantial 8% reduction in application duration (95% CI = -0.15, -0.01, p = 0.025), as reported both in Table 3 and the accompanying text. No other effects of age were observed.

**Table 3:** Association of extraction and pulpectomy procedure with the intensity and duration of the iontophoresis group A multiple linear regression model was used to estimate the p-value.

	Current Intensity, mA		Duration of Application, time	
	Β-Coefficient (95% CI)	p-value	Β-Coefficient (95% CI)	p-value
Pulpectomy	-0.35(-0.81, 0.11)	0.133	0.35(0.03, 0.67)	0.034
Age	0.05(-0.05, 0.14)	0.345	-0.08(-0.15, -0.01)	0.025
Girls compared to boys	0.23(-0.21, 0.66)	0.307	0.01(-0.31, 0.33)	0.968

**Table 4:** Mean difference in intensity and duration of the procedure in the iontophoresis group Values suggesting intensity and duration of application for both procedures. Data are presented in mean ± SD compared by the independent t-test. *p<0.05 significant.

	Extraction n (%) n= 40	Pulpectomy n (%) n= 40	p-value
Current intensity (in mAmp)	9.43 ± 0.95	9.07 ± 1.34	0.27
Duration of application (in min)	1.85 ± 0.80	2.40 ± 0.74	0.03

**Figure 3:**
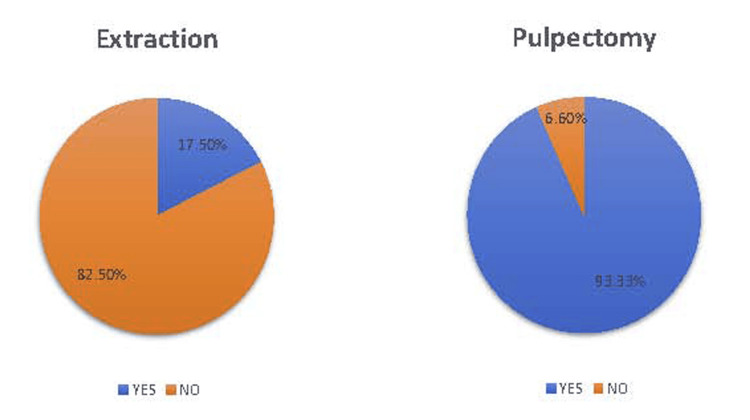
Need for additional LA for the individuals of Group A LA: Local anesthesia

Figure 3 illustrates the number of individuals who require local infiltration anesthesia after applying iontophoresis. For the extraction procedure, seven patients (17.5%) required additional local infiltration anesthesia; for the pulpectomy procedure, 14 patients (93.3%) required additional local infiltration anesthesia.

## Discussion

The present research demonstrated that for extraction, the mean value of current intensity was 9.43 + 0.95, and for the pulpectomy procedure, it was 9.07 + 1.34. The majority (81.8%) received the current intensity between 9 mA and 10 mA on the surface area of 4 cm^2^ (10 mA /4cm^2^-2.5 mA). Gangrosa et al. established the 0-3 mA/ cm^2^ current intensity required to extract deciduous teeth for 5 to 15 minutes following this study [6].

Direct current iontophoresis was shown to be more effective than alternating current iontophoresis in improving the distribution of lidocaine hydrochloride into and across the pig skin. This was done to improve the delivery of lidocaine hydrochloride [7]. Pain is typically caused by an excessive current density (Sanderson et al., 1989) [8], and a high current density combined with an extended application time would generate an extremely high pH, resulting in a chemical burn (Miller et al., 1987) [9]. In the current investigation, we did not observe any local or systemic adverse effects during or after iontophoresis administration; this was true for both the process and its aftermath.

**Figure 4:**
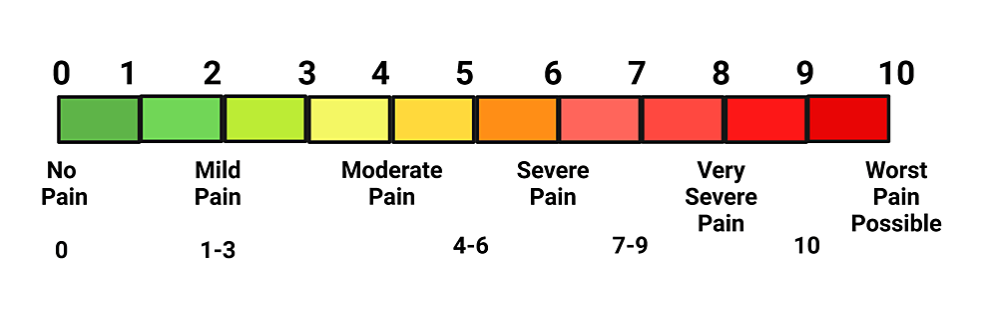
Schematic diagram of visual analog scale for pain evaluation. Notes: This figure has been drawn utilizing the premium version of BioRender with the License number YS25LQHYT0. Image Credit: Susmita Sinha

Participants in the study ranged in age from 5 to 12 years, with 8.5 years serving as the study's average participant age. Patients' reports of their discomfort level are frequently considered reliable and informative indicators of pain severity. Facial pain measures should be recommended over visual analog scales (Figure 4) to evaluate pain in young people since they are simpler to implement and more concrete. Since the participants in this study are now school-aged, it is possible to accurately quantify the levels of self-reported pain experienced by this age group because they are currently involved in concrete operations. The WBFPRS was utilized to meet the research inquiry and used successfully with individuals aged three years and older and is easy to use and comprehend. The scale was used since it is less expensive than other facial pain assessment scales and has high acceptability among children, parents, and medical professionals. Although it is crucial to keep in mind from a clinical perspective that children who are not cooperative may provide erroneous pain assessments, the children who were chosen for this study were all cooperative.

In Group A, out of 40 sites, the extraction procedure was completed successfully in 33 sites (82.5%), as deep topical anesthesia was achieved with iontophoresis for painless extraction. These findings are similar to those of Gangrosa et al., and current study data analysis suggests Iontophoresis's effectiveness was irrespective of the root length [6]. The variation in the individual’s pain perception may be one of the reasons for the observed ineffectiveness among the seven sites (17.5%) that required additional LA.

In the Group A pulpectomy procedure, 36 (93.3%) out of 40 sites required additional LA. There is variation in the anatomic topologies of nerve networks and the way systems interact [10]. Any deviation can result in a suboptimal anesthetic reaction. A specific nerve branch may be congenitally missing [11]. The rational anesthetic process may occasionally fail to achieve the intended anesthetic effect. An agent deposition around the accessory nerve branches may be required for total pulpal anesthesia. Primary teeth have less myelinated nerve fibers, which are lost early in the resorptive process [12]. This might make them less receptive to anesthesia, which could explain why iontophoresis in pulp treatment is less successful, needing further LA injection in this experiment [10]. Observed data revealed that the root length and number of roots are not the factors to predict the effectiveness of iontophoresis (Tables 5, 6). 

**Table 5:** Comparison of the number of teeth, mean root length, and effectiveness of Iontophoresis for extraction treatment from Group A(I). LA: Local anesthesia

Teeth Quadrant	Total Number of Teeth	Iontophoresis		Iontophoresis + Additional LA	
		Number of Teeth	Mean Root Length (in mm)	Number of Teeth	Mean Root Length (in mm)
Maxillary Anterior	8	6	9.3	2	4.4
Maxillary Posterior	14	13	4.7	1	4.5
Mandibular Anterior	1	1	9.8	0	-
Mandibular Posterior	17	13	4.6	4	6.1
Total	40	33	7.1	7	5.0

**Table 6:** Comparison of the number of teeth, mean root length, and effectiveness of iontophoresis for pulpectomy treatment from Group A(I). LA: Local anesthesia

Teeth Quadrant	Total Number of Teeth	Iontophoresis		Iontophoresis + Additional LA	
		Number of Teeth	Mean Root Length (in mm)	Number of Teeth	Mean Root Length (in mm)
Maxillary Anterior	4	0	-	4	7.0
Maxillary Posterior	14	0	8.3	14	8.5
Mandibular Anterior	2	0	-	2	7.2
Mandibular Posterior	20	4	-	16	9.0
Total	40	4	8.3	36	7.9

The intra-group comparison of WBFPRS for Group A showed that the patients who underwent extractions had lower scores (1.38 ± 0.46) compared to patients who underwent pulpectomy (3.53 ± 0.92), which was statistically highly significant with a p-value of <0.001. Thus, it demonstrates the greater efficacy of iontophoresis during extraction procedures. It has been explained as it ensures deeper penetration of dental tissues than the deep topical anesthetic infiltrating method, via iontophoresis, even in the hard palate, with minimal patient discomfort [6]. In Group B, patients undergoing the extraction procedure had a higher WBFPRS (3.83 ± 0.96) compared to patients undergoing pulpectomy (3.07 ± 1.34), which was statistically significant with a p-value of <0.04. Moreover, iontophoresis is a true needle-free modality for anesthesia. This study during subjective analysis found that iontophoresis was very well accepted among the pediatric patients compared to needle and syringe anesthesia, which itself provokes fear and anxiety. Many children with dental issues develop needle phobias. Consequently, they are reluctant to cooperate during the initial phase of the treatment. 

In the inter-group comparison of WBFPRS, scores were lower in Group A compared to Group B, which was statistically highly significant with a p-value of <0.001. Hence, iontophoresis was demonstrated as a successful, non-invasive, and less painful technique for deep topical anesthesia. The present study result agrees with the study performed by Gangarosa et al. (1974) [6].

**Figure 5:**
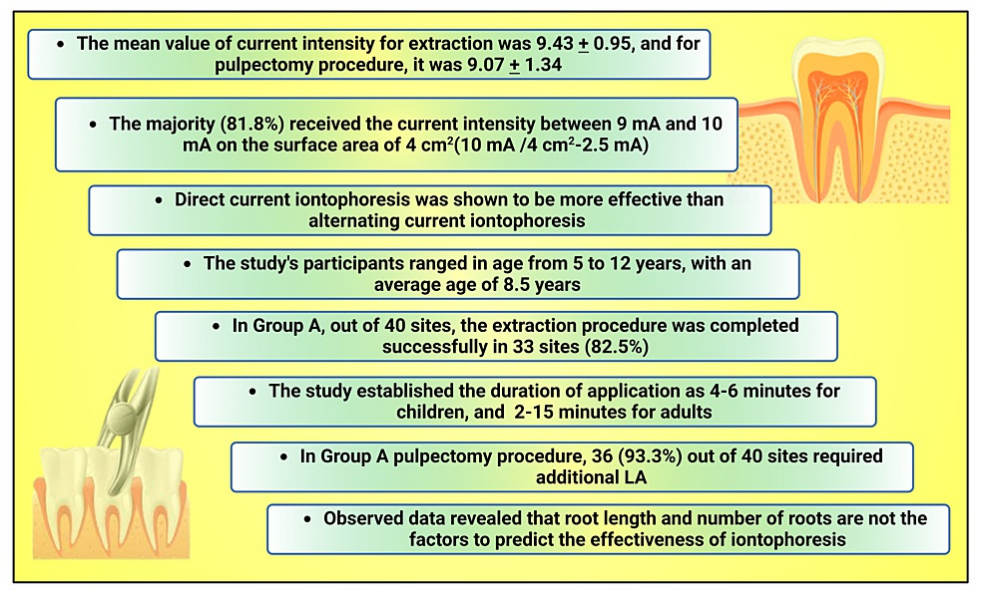
Chart illustrating study highlights This figure has been drawn utilizing the premium version of BioRender with license number JQ25MV4CTM. Image Credit: Susmita Sinha LA: Local anesthesia

The current study is a forerunner to show the application of the iontophoresis procedure for more invasive treatments like extraction and pulpectomies. The study established the duration of application as 4-6 minutes for children, in agreement with the previous studies, which range from 2-15 minutes for adults [6,13] (Figure 5).

A potential limitation of this study would be the smaller sample adopted in the pulpectomy cases. Independent examiner evaluation of patient comfort and duration of post-treatment anesthesia effects with Iontophoresis was crucial to improved inferences. This study evaluates patient comfort and duration of anesthesia. These two issues were evaluated by separate researchers. Ongoing clinical trials adopting a modified application of the Iontophoresis technique for pulpectomies may yield positive outcomes given the latest data procured.

Limitations of the study

The sample size and single-center research were the major limitations of the current study. Age and sex were the principal confounders of the present research as the details of these two factors were gathered during the data collection phase. After that, age and sex factors were incorporated as covariates in the regression model to mitigate the influence of confounding variables. Since no additional sociodemographic data were obtained, we are unable to examine the impacts of other independent factors. As a result, we have designed another randomized control multi-center clinical trial with more significant sample numbers and a longer duration so that the study outcomes may be generalized to the entire country. In the current study, clinical parameters and treatment approaches were conducted by a single investigator. Because this experiment was not double-anonymized, data collection may have been biased. As a result, future research could benefit from double blinding to avoid an opinionated introduction.

## Conclusions

This study suggested that the current intensity was 9.43 + 0.95 mA and a duration of 4 to 6 min for applying LA via iontophoresis to achieve the desired therapeutic level for dental surgery. Deep topical anesthesia achieved with the topical anesthetic spray infused by ionophoresis showed a significant reduction in pain and anxiety associated with needle insertion among the study population. Iontophoresis acts as a beneficial adjunct in the treatment and management of pediatric dental patients. This study may serve as a springboard for future research into this new modality. Further research with a larger sample size is required to lay the foundations of the fact-based pharmacodynamics potential of this unique needle-free anesthesia so that pediatric and adult anxious and trypanophobia patients can undergo treatment with minimal anxiety and distress.

## Figures and Tables

**Figure 6:**
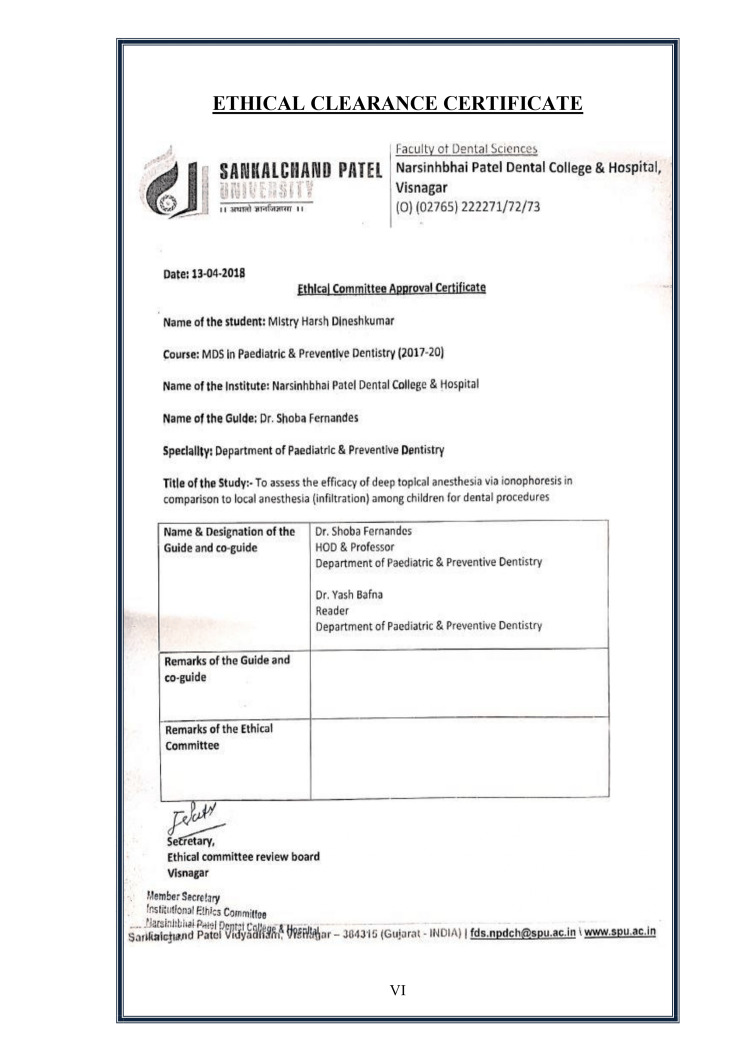
Ethical approval certificate Note: Appendix 1.
